# Supplementary material for: Validation of a single-step, single-tube reverse transcription loop-mediated isothermal amplification assay for rapid detection of SARS-CoV-2 RNA
Source: J Med Microbiol. 2020 Aug 5;69(9):1169–78. doi: 10.1099/jmm.0.001238 (PMC7656183; doi:10.1099/jmm.0.001238)
Supplement: Supplementary material 1 [file jmm-69-1169-s001.pdf]

Supplementary data

Supplementary Table S1. Results of N1-STOP-LAMP external quality assessment among four laboratories.

Sample	E-gene RT-qPCR Ct	N1-STOP-LAMP Tp				Agreement with RT-qPCR
		Lab A	Lab B	Lab C	Lab D	
1	16.3	06.31	08:00	05:12	04:48	4/4
2	nd	nd	nd	19:54	nd	3/4
3	16.5	06.54	04:26	06:06	04:54	4/4
4	nd	nd	nd	nd	nd	4/4
5	20.4	08.51	08:44	08:12	07:24	4/4
6	nd	nd	nd	nd	nd	4/4
7	21.7	09.30	05:58	07:12	06:54	4/4
8	nd	nd	nd	nd	nd	4/4
9	22.1	12.3	12:06	11:36	07:00	4/4
10	nd	nd	nd	nd	nd	4/4
11	22.8	08.45	05:18	07:24	07:54	4/4
12	25.4	16.45	16:16	09:30	07:06	4/4
13	25.9	24.30	26:21	20:48	25:12	4/4
14	27.2	nd	24:32	nd	nd	1/4
15	27.6	29.15	25:18	23:18	28:06	4/4
16	28.5	29.15	nd	nd	nd	1/4
17	29	nd	nd	nd	nd	0/4
18	30.6	29.15	23:04	nd	24:00	3/4
19	nd	nd	nd	nd	nd	4/4
20	nd	nd	nd	nd	nd	4/4

Ct = cycle threshold; Tp = time-to-positive

Company Limited by Guarantee I Registered in England No. 1039582 I Registered Office Charles Darwin House, 12 Roger Street, WC1N 2JU, UK
Registered as a Charity: 264017 (England & Wales); SC039250 (Scotland)

Supplementary Table S2. N1-STOP-LAMP screen of NATtrol™ Respiratory Panel 2 (RP2) Controls.

Virus/Organism	Strain	RP2 Control 1	RP2 Control 2	N1-STOP-LAMP
**Adenovirus Type 1**	N/A	Positive	Negative	Negative
**Adenovirus Type 3**	N/A	Positive	Negative	Negative
**Adenovirus Type 31**	N/A	Positive	Negative	Negative
C. pneumoniae	CWL-029	Positive	Negative	Negative
**Influenza A 2009 H1N1pdm**	A/NY/02/2009	Positive	Negative	Negative
**Influenza A 2009 H3N2**	A/Brisbane/10/07	Positive	Negative	Negative
**Human Metapneumovirus Type 8**	Peru6-2003	Positive	Negative	Negative
M. pneumoniae	M129	Positive	Negative	Negative
**Parainfluenza Type 1**	N/A	Positive	Negative	Negative
**Parainfluenza Type 4**	N/A	Positive	Negative	Negative
**Rhinovirus Type 1A**	N/A	Positive	Negative	Negative
B. parapertussis	A747	Negative	Positive	Negative
B. pertussis	A639	Negative	Positive	Negative
**Coronavirus 229E**	N/A	Negative	Positive	Negative
**Coronavirus HKU-1**	Recombinant	Negative	Positive	Negative
**Coronavirus NL63**	N/A	Negative	Positive	Negative
**Coronavirus OC43**	N/A	Negative	Positive	Negative
**Influenza A H1N1**	A/New Cal/20/99	Negative	Positive	Negative
**Influenza B**	B/Florida/02/06	Negative	Positive	Negative
**Parainfluenza Type 2**	N/A	Negative	Positive	Negative
**Parainfluenza Type 3**	N/A	Negative	Positive	Negative
**RSV Type A**	2006 isolate	Negative	Positive	Negative

Table S3. Clinical data positivity summary (107 E-gene RT-qPCR positives vs N1-STOP-LAMP Tp).

Sample	E-gene RT-qPCR Ct	N1-STOP-LAMP Tp (min:sec)	N1-STOP-LAMP retest of RNA
1	23.0	17:45	
2	21.0	08:05	
3	23.0	09:45	
4	26.0	28:00	
5	nd	nd	
6	21.2	11:00	
7	17.8	10:00	
8	16.3	08:15	
9	19.3	08:45	
10	18.6	08:30	
11	25.1	20:00	
12	22.0	13:45	
13	28.1	14:00	
14	23.2	11:00	
15	24.3	20:15	
16	28.3	29:15	
17	23.1	17:30	
18	26.1	28:15	
19	16.4	07:45	
20	20.9	11:45	
21	27.2	16:45	
22	20.1	10:15	
23	20.4	11:00	
24	20.0	08:45	
25	18.9	09:00	
26	21.6	11:45	
27	27.1	27:00	
28	21.9	16:00	
29	17.0	09:45	
30	15.4	09:00	
31	19.3	17:15	
32	20.6	11:15	
33	15.1	09:30	
34	27.6	20:30	
35	28.5	21:45	

36	16.3	09:15	
37	16.5	09:45	
38	23.1	22:30	
39	31.5	nd	
40	30.5	nd	
41	25.9	23:15	
42	21.3	13:30	
43	30.6	29:15	
44	25.4	15:45	
45	22.8	13:15	
46	27.2	29:15	
47	21.7	08:30	
48	16.5	07:15	
49	20.4	08:30	
50	21.8	10:15	
51	24.4	12:00	
52	15.1	08:00	
53	28.8	27:00	
54	18.2	07:45	
55	17.2	06:00	
56	26.7	09:00	
57	23.9	11:30	
58	16.2	06:30	
59	17.8	07:15	
60	27.8	24:15	
61	27.2	11:45	
62	35.8	nd	
63	22.8	11:45	
64	17.0	08:00	
65	20.0	16:45	
66	19.5	08:45	
67	24.8	12:15	
68	29.2	28:30	
69	32.3	nd	
70	26.6	20:30	
71	33.1	28:00	
72	20.0	07:30	
73	18.6	08:30	
74	17.5	07:50	
75	30.7	25:30	
76	20.9	13:00	
77	19.0	07:15	

78	26.3	11:45	
79	22.6	08:30	
80	29.3	nd	
81	20.9	08:30	
82	21.5	14:15	
83	20.0	07:45	
84	26.6	nd	
85	28.5	nd	
86	28.2	26:15	
87	30.3	nd	
88	27.6	13:15	
89	20.0	09:15	
90	27.4	27:45	
91	18.7	07:15	
92	30.0	20.30	
93	28.0	nd	10:00
94	33.2	nd	nd
95	19.7	08:45	
96	28.2	nd	17:45
97	34.7	09:00	
98	25.8	nd	25:30
99	21.3	12:15	
100	16.5	07:45	
101	33.4	25:15	
102	25.1	19:00	
103	20.3	09:45	
104	26.8	nd	12:25
105	25.1	16:00	
106	22.4	12:30	
107	21.4	08:25	

Ct = cycle threshold; Tp = time-to-positive
